# Book Reviews

**Published:** 1918-03

**Authors:** 


### BOOK REVIEWS

Books mentioned may be inspected at and ordered through this office. So far as possible, books received in any month will be reviewed in the issue of the second month following. Pamphlets, quarterly and similar periodicals, reports, transactions, etc., will, as a rule, merely be mentioned.

---

**Food For the Sick—A Manual for the Physician and Patient.** By Solomon Strouse, M. D., and Maude A. Perry, A. B. W. B. Saunders Company, Philadelphia and London, 1917. Price $1.50.

The first part of the book gives a concise review of the general principles of nutrition and dietetics, with good reference lists of the chemical composition and caloric values of foods. Diseases of the various systems of the body are next taken up in order, the physiology and pathology of each condition given as far as it relates to indications for diet, and detailed menus are given with the principles involved. I was interested on the chapter on diabetes as it is practical and should be of great practical service to men treating such cases. Particularly good is the list of foods arranged according to their percentage composition of carbohydrates, but all the articles are useful and the book is both valuable and practical.

---

**Physical Diagnosis.** Part 1 by George William Morris, A. B., M. D., Associate in Medicine at the University of Pennsylvania. Part II. by H. R. M. Landis, A. B., M. D., Director of Clinical and Sociological Department of the Phipps Institute, Philadelphia. Octavo of 1,000 pages with 325 illustrations, mostly original. W. B. Saunders Company. Cloth $7.00 net.

**Diagnostic Et Traitement De La Meningite Cerebro-Spinale.** Dr. Dopter, Prof. at the School of Val-de-Grace, 96 pages, 17 illustrations, 2 francs. J-B. Bailliere et Fils, 19, rue Hauteville, Paris.

This is one of the series of Actualites Medicales.

---

**Physical Diagnosis.** By W. D. Rose, M. D., Associate Professor of Medicine and Lecturer on Physical Diagnosis in the Medical Department of the University of Arkansas. 500 pages, 6x9, with 294 illustrations. Cloth, $4.00. St. Louis, C. V. Mosby Co., 1917.

Dr. Rose has produced a handsome book in which the text is amply supplemented by pictorial and diagrammatic illustrations, taken mostly from standard works. In addition to physical diagnosis proper, he gives us about one hundred pages on "the principal diagnostic signs referable to the head, neck, and limbs, together with a minimum examination of the nervous system." The descriptive matter is clear and well arranged and a copious index makes every page readily available for reference. The book is admirably adapted to the use of the practitioner who wants guidance in diagnosis without an excessive amount of discussion over the finer points on which authorities are apt to differ. Thus we note that roentgenography is very lightly touched upon, because the author believes in exhausting the possibilities of careful examination and clinical study before resorting to the application of the X-rays.

---

**Technic of the Irrigation Treatment of Wounds by the Carrel Method** by J. Dumas and Anne Carrel. Authorized translation by Adrian V. S. Lambert, M. D., Acting Professor of Surgery in the College of Physicians and Surgeons (Columbia University), New York. With an Introduction by W. W. Keen, M. D., L.L.D., F. R. C. S. (Hon.). Cloth, 90 pages, illustrated. Price, $1.25. Paul B. Hoeber, New York, 1917

This little book was prepared by Madame Carrel and Dr. Dumas to explain and illustrate the details of the Carrel method. It is designed especially for the use of nurses and assistants, and to supplement the more technical treatment of the subject by Dr. Carrel. All the essentials of the method are given in compact form without any admixture of surgical or pathological details. There is a very serviceable glossary of the French and English terms pertaining to the technic.

Dr. Keen says: "Every surgeon in the various military and naval forces and also those in civil life who have to do with industrial and other accidental wounds should know this technic by heart and practice it with exactness. They will be rewarded by a most gratifying success."

---

**The Modern Gasoline Automobile: Its Design, Construction, Operation and Maintenance.** A Practical, Comprehensive Treatise Explaining All Principles Pertaining to Gasoline Automobiles and Their Component Parts. The Most Complete Up-to-Date Treatise on Gasoline Automobiles Ever Published. By Victor W. Page, M. E. Seventh Edition. Cloth. Price, $3 net. Pp. 1032, with 100 illustrations. New York: The Norman W. Henley Publishing Company, 1918.

This book is described as "a liberal education in the automobile art." It is nearly twice as large as the original edition, published six years ago. Physicians will appreciate the fact that it is a complete treatise on the anatomy and physiology of the automobile, solving many of the mysteries that perplex the buyer and operator of this modern mechanical wonder.

---

**Progressive Medicine,** Vol. XX, No. 4.. Edited by Hobart Amory Hare, M. D., Professor of Therapeutics, Materia Medica and Diagnosis in the Jefferson Medical College, Philadelphia; assisted by Leighton F. Appleman, M. D., Instructor in Therapeutics, Jefferson Medical College, Philadelphia. December 1, 1917. Octavo, 466 pages, illustrated. Quarterly, $6.00 per annum. Philadelphia, Lea & Febiger.

This number reviews recent progress in our knowledge of diseases affecting the digestive tract and allied organs, the kidneys, and the genito-urinary organs. One chapter is devoted to military surgery, and another to practical therapeutic referendum. The high standard of this well known publication is maintained in these articles.

---

**The Medical Clinics of North America.** Volume I, Number III, (The New York Number, November, 1917). Octavo of 346 pages, 37 illustrations. Philadelphia and London: W. B. Saunders Company. Published Bi-monthly. Price, per year: Paper, $10.00; Cloth, $14.00.

This volume brings together the clinical teachings of a score or more of the leading authorities on internal medicine in New York City, representing seven hospitals and medical schools. The cases selected for discussion represent conditions that the general practitioner is likely to meet in his every day experiences. The method of presentation gives the reader a good idea of the latest advances in case-teaching as demonstrated in our leading institutions. As a source of practical information on the subjects considered the volume could hardly be improved.

---

**Treasury Annual Reports, 1917, U. S. Public Service.**

---

**Proceedings of the Eleventh Annual Meeting,** Association of Life Insurance Presidents, New York, Dec. 6-7, 1917.

---

**Bureau of American Ethnology,** Bulletin 63.

